# Meaning in Life in Late-Stage Parkinson’s Disease: Results from the Care of Late-Stage Parkinsonism Study (CLaSP) in Six European Countries

**DOI:** 10.1007/s10943-023-01962-w

**Published:** 2023-12-05

**Authors:** Sarah K. Bublitz, Cornelia Brandstötter, Martin Fegg, Joaquim J. Ferreira, Per Odin, Bastiaan R. Bloem, Wassilios G. Meissner, Richard Dodel, Anette Schrag, Stefan Lorenzl

**Affiliations:** 1 Institute of Palliative Care, Paracelsus Medical University in Salzburg, Strubergasse 21, 5020 Salzburg, Austria; 2 Krankenhaus Agatharied, Norbert-Kerkel – Platz, 83734 Hausham, Germany; 3 Psychotherapeutic Practice, Munich, Germany; 4 Faculty of Medicine, Molecular Medicine Institute, The University of Lisbon, Lisbon, Portugal; 5 Division of Neurology, Department of Clinical Sciences, Lund University, Skane University Hospital, Lund, Sweden; 6 Department of Neurology, Centre of Expertise for Parkinson and Movement Disorders, Donders Institute for Brain, Cognition and Behaviour, Radboud University Medical Centre, Nijmegen, The Netherlands; 7 CHU Bordeaux, Service de Neurologie des Maladies Neurodégénératives, IMNc, CRMR AMS, NS-Park/FCRIN Network, 33000 Bordeaux, France; 8 Univ. Bordeaux, CNRS, IMN, UMR5293, 33000 Bordeaux, France; 9 Department Medicine, University of Otago, Christchurch, and New Zealand Brain Research Institute, Christchurch, New Zealand; 10 Department of Geriatric Medicine, University Hospital Essen, Essen, Germany; 11 Queen Square Institute of Neurology, University College London, London, UK; 12 Interdisciplinary Center for Palliative Medicine and Clinic for Neurology, University of Munich, Munich, Germany

**Keywords:** Late-stage Parkinson’s disease, Meaning in life, Coping, Response shift

## Abstract

The Care of Late-Stage Parkinsonism (CLaSP) study is a longitudinal, multicentre, prospective cohort study to assess the needs and provision of care for people with late-stage Parkinson’s disease and their caregivers in six European countries. As a cross-sectional study within the CLaSP study, 509 people with Parkinson’s disease completed the “Schedule-for-Meaning-in-Life-Evaluation” (SMiLE) questionnaire. We compared the results to those of a representative sample of healthy participants (*n* = 856). People with late-stage Parkinson’s disease reported family, partnership and spirituality as the greatest areas of importance. Overall, they had lower SMiLE indices compared to healthy participants. People with late-stage Parkinson’s disease rated the importance of core meaning in life areas (namely family, social relations and health) as significantly lower than the representative cohort and they also rated satisfaction as significantly lower in most areas. In conclusion, people with late-stage Parkinson’s disease do have areas where they can find meaning, such as family, partnership and spirituality. However, they indicate a lack of fulfilment of their individual MiL, reflected by low satisfaction rates in the majority of meaning in life categories. The need for spiritual support for people with Parkinson’s disease indicates the important role of chaplains to help people with Parkinson’s disease maintain meaning in life.

## Introduction

Parkinson’s disease causes increasing disability with disease progression and leads to high levels of burden for relatives and other informal caregivers. Substantial changes in behavioural and psychological symptoms can contribute to the burden to both patients and their carers (Aarsland et al., 1999; Barone et al., 2009). Research has mainly focused on quality of life in Parkinson’s disease, but meaning in life has not been explored in detail yet. In the European context, the study of meaning in life is closely related to research on existential spiritual care in healthcare setting. In German (*Lebenssinn, Lebenssinn finden*), the concept of meaning in life is closely related to Victor Frankl’s idea of sense-making in human suffering. In Dutch, the study of spiritual care is literally called *Sinnstiftungsforschung* (*Zingeving*). The construct of meaning in life has generated increasing interest among clinicians and researchers in other areas of palliative care (Breitbart et al., 2010; Chochinov et al., 2011; Lee et al., 2006).

In late-stage Parkinson’s disease, one of the main challenges for clinicians and allied professionals is to help people to cope with meaninglessness in suffering, which prevails despite multi-dimensional symptom control and interdisciplinary support. Wishes to hasten death can be the result of this suffering. Meaning in life is highly individual and varies in different situations (Auhagen, 2000). It seems to serve as a safeguard against depression, hopelessness and desire for hastened death among terminally ill oncological patients (Breitbart et al., 2000). Furthermore, meaning in life is significantly and negatively associated with psychological distress (Bernard et al., 2017) and acts as an important contributor to quality of life in palliative care patients and the general population (Bernard et al., 2020).

The “Schedule for Meaning in Life Evaluation” (SMiLE) (Fegg et al., 2008) is an instrument developed for the assessment and description of individual meaning in life. It assesses the content of the reported meaning as well as a rating of importance (weighting) and level of satisfaction with each meaning in life area. The SMiLE has been validated in cancer and non-cancer palliative care patients (Fegg et al., 2010a, 2010b; Stiefel et al., 2008), including patients with progressive supranuclear palsy (PSP), an atypical Parkinsonian disorder (Fegg et al., 2014). In PSP patients, data have shown that with decreasing physical condition, the areas that provide meaning shift away from health and more towards other areas. PSP patients find meaning in family and close relationships, nature/animals and home/garden, which could be explained as a coping mechanism referred to as “response shift”. This has also been observed in patients with amyotrophic lateral sclerosis (Fegg et al., 2010b) and palliative care patients (Fegg et al., 2010a).

This study addresses meaning in life in people with late-stage Parkinson’s disease, a group that is faced with considerable disease severity and burden. It is the first increasingly atheist and non-religious large-cohort study from six European countries that focuses on meaning giving aspects of life, which can be helpful to all professionals involved in providing care for people with Parkinson’s disease.

## Objectives

The aim of this study was to investigate meaning in life in people with advanced-stage Parkinson’s disease in a large, cross-sectional, multicentre cohort and compare these findings with data from a representative sample of the German population (Fegg et al., 2007). Using the SMiLE, we aimed to evaluate and categorize individually important meaning in life areas and to examine differences between people with advanced Parkinson’s disease and healthy individuals.

## Methods

### Study Design

The CLaSP study is a multicentre cohort study of people with late-stage Parkinson’s disease and their caregivers in six European countries (London and Luton, UK; Marburg-Giessen, Essen, and Munich, Germany; Nijmegen, The Netherlands; Bordeaux, France; Lisbon, Portugal; and Lund, Sweden). Late-stage Parkinson’s disease was defined as Hoehn and Yahr (HY) stages IV to V (score range 1–5, higher = worse) (Hoehn & Yahr, 1967) while on medication and/or having a substantial need of help with Activities of Daily Living (ADL). The CLaSP study design has been described in detail elsewhere (Balzer-Geldsetzer et al., 2018). The CLaSP study particularly aimed to include patients who were not under regular specialist follow-up. The goal of recruitment was to establish a large European cohort with as many patients as possible. Therefore, patients were identified through various health care settings at the different centres: neurology departments; the municipality-based health care system; and care of the elderly, palliative care, and primary care settings.

### Assessments

Participants were assessed at baseline using a range of scales and questionnaires, including the Schedule for Meaning in Life Evaluation (SMiLE), an individualized measure of meaning in life.

Initially, it was planned to assess meaning in life using the SMiLE in a longitudinal manner, but response rates at T2 (6 months) and T3 (12 months) were scarce and therefore it was decided to conduct a cross-sectional study design regarding the analysis of SMiLE.

Respondents were asked to list up to seven areas important for their meaning in life and then to rate the current level of satisfaction with and importance of each area. The level of satisfaction with each area is rated on a seven-point Likert scale, ranging from -3 “very unsatisfied” to + 3 “very satisfied”. The importance of each area is rated with an eight-point scale, ranging from 0 “unimportant” to 7 “extremely important”. The Index of Satisfaction (IoS) is calculated as the mean score of satisfaction or dissatisfaction with the individual meaning in life areas; the Index of Weighting (IoW) indicates the mean score of importance of the meaning in life areas. In the total SMiLE index (Index of Weighted Satisfaction, IoWS), the ratings for importance and satisfaction are combined (all ranges, 0–100, with higher scores reflecting higher meaning in life). Calculation of indices is reported elsewhere (Fegg et al., 2008).

For analysis, areas of importance to meaning in life by an individual are classified in categories. Originally, 13 categories for individual meaning in life were identified by cluster analyses (Fegg et al., 2007). Since then, the categories “Art/Culture” and “Growth” were added for providing a more precise categorization. The areas of meaning in life listed by people with Parkinson’s disease in our cohort were each assigned to one of the 15 categories, according to the current version of the SMiLE manual (https://www.psychotherapie-muenchen.de/downloads/SMiLE_Manual.pdf).

Other assessments of patients and carers within the CLaSP cohort study include standardized questionnaires to evaluate disease severity, comorbidities, depression, cognition, non-motor symptoms, quality of life in patients and carers as well as caregiver burden.

Data from the CLaSP cohort were compared to a representative sample of 856 healthy German participants from all age groups (12.9% were age 70 and above) (Fegg et al., 2007).

### Statistical Analysis

Student’s *t* test was used to compare the number of meaning in life areas listed in each group. Differences were considered to be statistically significant at *p* < 0.05 and were then Bonferroni corrected.

To identify variables that influence the Index of Weighted Satisfaction (IoWS), the categories age, age at onset, marital status, gender and trial site were entered into a stepwise regression model as potentially confounding variables. Since all variables except trial site were omitted in this model as being of non-significant influence, a linear regression modelling for trial site as the variable was calculated against the dependent variable Index of Weighted Satisfaction (IoWS). For this model, the unstandardized regression coefficient (*B*), its standard deviation, the standardized coefficient (*B*), the total explained variance (*R*^2^) and its respective *P* value are presented.

Statistical tests were performed using the Statistical Package for Social Sciences (SPSS Version 25) and Excel.

In the comparative study in healthy participants (Fegg et al., 2007), the original version of the SMiLE was used where the importance of each area was rated with a five-point adjectival scale, ranging from 1 ‘somewhat important’ to 5 ‘extremely important’. To adjust this to the newer version using the eight-point scale from 0 ‘not important’ to 7 ‘extremely important’, the original data were adjusted (*W*/5 * 7).

### Ethical Approval

The CLaSP study is being conducted in compliance with the Helsinki Declaration (World Medical Association Declaration of Helsinki 1997), i.e. detailed oral and written information was given to study participants and their informants to ensure that they fully understand potential risks and benefits of the study. The study was approved by the ethical review board of each individual centre. Written informed consent was obtained from all participants. In case the patients were unable to sign, consent was given by the legal representative, mostly a spouse or family member, in accordance with the country-specific legal requirements.

## Results

### Participation in the Study

692 people with Parkinson’s disease fulfilled the inclusion criteria and were included in the CLaSP study. At Baseline (T1), SMiLE questionnaires were distributed to people in 8 centres and we received the completed SMiLE from 509 participants (74% of all CLaSP study participants). Table 1 shows the number of responses from each centre.

**Table 1:** Total number of participants and completion of SMiLE from each of the eight centres

Centre	Distributed questionnaires T1	Completed	Response rate (%)
1	147	84	57
2	81	74	91
3	108	60	56
4	77	52	68
5	105	82	78
6	83	51	61
7	120	91	75
8	27	15	56

*Participants’ characteristics* of the CLaSP study are described in detail elsewhere (Schrag et al., 2020). Average disease duration in the CLaSP cohort was 15.4 (SD 7.7) years, and mean total UPDRS score was 82.7 (SD 22.4). Some demographic data of participants of the SMiLE study within CLaSP are presented in Table 2.

**Table 2:** Demographic data of participants (*n* = 509)

Gender	45% female 55% male
Country (number of patients; % of all participants)	UK: 84 (17%) Germany: 180 (35%) Portugal: 60 (12%) France: 52 (10%) Sweden: 82 (16%) The Netherlands: 51 (10%)
Age at study inclusion (years; mean, standard deviation)	75.4 (± 8.2)
Age at onset (years; mean, standard deviation)	60.3 (± 12.4)
Disease duration (years; mean/standard deviation)	15.1 (± 9.1)

#### Categories of Meaning in Life

In total, people with Parkinson’s disease listed 1596 areas contributing to personal meaning in life (mean 3.1 areas per participant). All listings were assigned to the categories derived from the representative survey (Fegg et al., 2007) and according to the current SMiLE manual (https://www.psychotherapie-muenchen.de/downloads/SMiLE_Manual.pdf), which now lists 15 categories. Comparison with the representative cohort is therefore not available for all meaning in life areas.

Table 3 presents percentages of participants in both samples who listed individual meaning in life areas in the respective category along with means and standard deviations of the satisfaction and importance ratings. People with Parkinson’s disease described fewer areas of meaning in life (2.3 vs 5.5 on average). They most commonly listed family, leisure time and friend/social relations as areas giving meaning in life. They listed work (6.1% vs 54.1%), finances (3.3% vs 14.5%) and health (12.2% vs 32.2%) less often than respondents in the reference group but animals/nature (8.8% vs 9.1%) and leisure time (38.9% vs 40.9%) in similar frequency to the control group.

**Table 3:** Areas of meaning in life

Areas of MiL	Late-Stage Parkinson (*n* = 509)			Reference group (*n* = 856)			*t* test	*t* test
	*N* (%)	Importance (*w*, range 0 to 7) mean** +/− **SD	Satisfaction (*s*, range − 3 to + 3) mean** +/− **SD	*N* (%)	Importance (*w*, range 0 to 7) mean** +/− **SD	Satisfaction (*s*, range − 3 to + 3) mean** +/− **SD	*p* Value importance	*p* Value satisfaction
Family	334 (65.6%)	**6.1 +/− 2.0**	**1.9 +/− 1.8**	708 (82.7%)	**6.6 +/+ 1.0**	**2.3 +/− 0.9**	**< 0.01**	**< 0.01**
Partnership	91 (17.9%)	6.4** +/− **1.0	2.0** +/− **1.7	233 (27.2%)	6.6 +/+ 0.9	2.4** +/− **1.1	0.04	0.01
Friends/social relations	136 (26.7%)	**5.5 +/− 1.8**	**1.1 +/− 1.8**	340 (39.7%)	**6.0 +/+ 1.1**	**2.2 +/− 1.0**	**< 0.01**	**< 0.01**
Work	31 (6.1%)	**4.4 +/− 1.8**	**0.1 +/− 2.1**	463 (54.1%)	**5.4 +/− 1.4**	**1.4 +/− 1.6**	**< 0.01**	**< 0.01**
Leisure time	198 (38.9%)	4.6** +/− **2.3	**0.3 +/− 2.0**	350 (40.9%)	4.8 +/+ 1.5	**1.6 +/− 1.4**	0.11	**< 0.01**
Home/garden	87 (17.1%)	4.9** +/− **2.1	**0.9 +/− 2.0**	81 (9.5%)	4.9** +/− **1.5	**2.0 +/− 1.1**	0.50	**< 0.01**
Finances	17 (3.3%)	5.5** +/− **1.3	0.4** +/− **2.1	124 (14.5%)	5.0 +/+ 1.5	1.0** +/− **1.8	0.15	0.26
Spirituality	31 (6.1%)	6.4** +/− **1.2	2.3** +/− **1.2	80 (9.4%)	6.1 +/+ 1.4	2.4** +/− **0.9	0.26	0.32
Health	62 (12.2%)	**5.8 +/− 2.3**	− **0.9 +/− 2.0**	276 (32.2%)	**6.7 +/− 0.8**	**1.8 +/− 1.5**	**< 0.01**	**< 0.01**
Satisfaction	13 (2.6%)	6.2** +/− **1.0	1.2** +/− **1.7					
Animals/Nature	41 (8.1%)	5.5** +/− **1.2	**0.8 +/− 1.9**	79 (9.2%)	5.8 +/+ 1.3	**2.3 +/− 1.0**	0.21	**< 0.01**
Altruism/social commitment	9 (1.8%)	5.3** +/− **1.1	1.6** +/− **1.9	39 (4.6%)	5.2 +/+ 1.2	2.1** +/− **0.9	0.81	0.45
Hedonism	30 (5.9%)	4.3** +/− **2.8	0.1** +/− **3.3					
Art/Culture	99 (19.4%)	5.0** +/− **1.5	0.9** +/− **1.9					
Growth	38 (7.5%)	5.4** +/− **2.7	0.6** +/− **2.0					
Well-being				37 (4.3%)	4.4 +/+ 0.8	1.8 +/+ 1.3		

Percentages of respondents listing each category, means (*M*) and standard deviations (SD) of the satisfaction (s, range − 3 to + 3), and importance ratings (*w*, range 0 to 7). Reference data from a representative sample of the German population (Fegg et al., 2007). Post-priori to the analyses of this representative study, the original 13 categories were defined. In the current SMiLE manual, these were extended to 15 categories (https://www.psychotherapie-muenchen.de/downloads/SMiLE_Manual.pdf)

*MiL* meaning in life, *SD* standard deviation

Bold values show significance after Bonferroni correction (significance level < 0.0017)

#### Importance of Meaning in Life Areas (Table 3)

Areas with the highest importance rates (> 6.0 on eight-point Likert scale between 0 and 7) for people with Parkinson’s disease were family, partnership, spirituality and satisfaction. The lowest importance was attributed to work and hedonism. Compared to the healthy reference group, the following meaning in life areas were of significantly lower importance to people with Parkinson’s disease: family (6.1 vs 6.6), social relations (5.5 vs 6.0), work (4.4 vs 5.4) and health (5.8 vs 6.7), and no area was given a significantly higher importance rating than the control group.

#### Satisfaction with Meaning in Life Areas (Table 3)

People with Parkinson’s disease stated lowest satisfaction (on average dissatisfied) in the areas health, hedonism and work. Compared to the healthy reference group, satisfaction was significantly lower for people with Parkinson’s disease in 7 of 11 comparable meaning in life areas: family (1.9 vs 2.3), social relations (1.1 vs 2.2), work (0.1 vs 1.4), leisure time (0.3 vs 1.6), home/garden (0.9 vs 2.0), health (− 0.9 vs 1.8) and animals/nature (0.8 vs 2.3).

### Meaning in Life in Parkinson’s Disease Patients—SMiLE Indices

In people with Parkinson’s disease, the indices of satisfaction (IoS), of weighting (importance) (IoW) and of weighted satisfaction (IoWS) were significantly lower than in the control group (mean IoS 65.6, mean IoW 79.4, mean IoWS 66.9, all *p* values < 0.01), as can be seen in Table 4. Data of 18 people with Parkinson’s disease were partially incomplete in either importance or satisfaction ratings; therefore, indices could not be calculated for these individuals (*n* = 491).

**Table 4:** SMiLE indices in people with late-stage Parkinson’s disease and in the representative sample German population (Fegg et al., 2007)

	CLaSP study/Parkinson *N* = 491	Representative study/healthy individuals *N* = 856	*t* test	ALS (Fegg et al., 2010b)	PSP (Fegg et al., 2014)	Palliative Care (Fegg et al., 2010a)
	Mean** +/− **SD	Mean** +/− **SD	*p* Value	Mean** +/− **SD	Mean** +/− **SD	Mean** +/− **SD
Index of Satisfaction	65.6** +/− **28.2	82.8 +/+ 14.7	< 0.01	74.7** +/− **20.2	68.6** +/− **25.6	70.2,** +/− **19.7
Index of Weighting	79.4** +/− **16.7	85.6 +/+ 12.3	< 0.01	88.1,** +/− **10.1	79.6** +/− **12.6	84.7,** +/− **11.5
Index of Weighted Satisfaction	66.9** +/− **28.3	83.3** +/− **14.8	< 0.01	76.3** +/− **20.5	69.2** +/− **26.1	72.0,** +/− **19.4

18 patient data sets of people with Parkinson’s disease had to be excluded for index calculation because of incomplete data in satisfaction or importance ratings

SMiLE indices for different patient groups reported in the literature (Fegg et al., )

*PD* Parkinson’s disease, *ALS* amyotrophic lateral sclerosis, *PSP* progressive supranuclear palsy

SMiLE indices of people with late-stage Parkinson’s disease are also lower than those reported for people with amyotrophic lateral sclerosis (ALS) (Fegg et al., 2010b), progressive supranuclear palsy (PSP) patients (Fegg et al., 2014) and palliative care patients (Fegg et al., 2010a) (see Table 4).

#### Regression Model on Variables on IoWS

In a stepwise regression model using the variables current age, age at onset, marital status, gender and trial site, the variable trial site was the only one to show significant influence on the Index of Weighted Satisfaction (IoWS) (see Tables 5 and 6). According to this calculation, the impact of trial site is, however, only marginal, which is reflected by a low Beta coefficient of − 0.132. Furthermore, the variable trial site only explains 1.5% of the variance of IoWS (reflected by an adjusted *R*^2^ of 0.015).

**Table 5:** Linear model on the influence of trial site on the Index of Weighted Satisfaction (IoWS) in people with late-stage Parkinson’s disease

	Unstandardized coefficient *B* (trial site)	Standard error (*B*)	Standardized coefficient *B*	Adjusted *R*^2^	*p* Value
IoWS (dependent variable)	− 1.731	0.591	− 0.132	0.015	0.004

**Table 6:** Standard linear regression model on variables age, age at onset, gender and marital status on the dependent variable IoWS in people with late-stage Parkinson’s disease

	Standardized coefficient (*B*)	*p* Value
Age	− 0.009	0.846
Age at onset	− 0.055	0.225
Gender	0.057	0.208
Marital status	0.045	0.316

All of these variables show no significant effect on IoWS

## Discussion

The CLaSP study is the first cohort study specifically addressing the clinical features, health care and spiritual needs, treatment strategies and outcomes of people with late-stage Parkinson’s disease in multiple centres across Europe. With the prevalence of Parkinson’s disease expected to rise in the coming years (Dorsey & Bloem, 2018), adequate comprehensive care including spiritual support in suffering and health-related loss of meaning in life for these patients will become a major challenge.

As part of this cohort study, meaning in life was assessed using the validated SMiLE instrument and was compared to a representative sample of the German population. In our cohort, the highest importance was attributed to family, partnership, spirituality and satisfaction, whereas understandably, the lowest importance was attributed to work and hedonism. Similarly to studies with palliative care patients (Fegg et al., 2010a) and patients with amyotrophic lateral sclerosis (Fegg et al., 2010b), people with late-stage Parkinson’s disease are more likely to list categories such as family and partner, friends, leisure time and home/garden and are less likely to prioritize health, finances and work. This response shift is considered to be a coping mechanism in people facing a life-limiting disease. However, importance ratings for family and social relations, as well as health and work, were also significantly lower for people with Parkinson’s disease than in the reference cohort. Even more remarkable are the differences in satisfaction with meaning in life areas for people with Parkinson’s disease. Satisfaction ratings are significantly lower in most of the comparable categories, even in areas that were given the highest importance ratings. Areas with the least satisfaction for people with Parkinson’s disease are health, work and hedonism.

Testing for the influence of demographic variables such as current age, age at disease onset, gender and marital status showed no significant influence on the Index of Weighted Satisfaction (IoWS) in our cohort of people with late-stage Parkinson’s disease. The only variable to show significant, yet still marginal, influence on the IoWS was the variable trial site, which attributes to 1.5% of IoWS variance.

Analysis of the SMiLE indices also indicates differences to other patient groups. The indices of satisfaction, weighting and weighted satisfaction are lowest in people with late-stage Parkinson’s disease, even when compared indirectly to previously published findings in other groups of patients with progressive and fatal diseases like amyotrophic lateral sclerosis (ALS), progressive supranuclear palsy (PSP) and palliative care patients. An explanation for the low indices might be the different disease duration which may affect coping strategies. In ALS as well as in palliative care, patients’ life expectancy is shorter compared to people with Parkinson’s disease in whom the late and even the palliative phase of the disease can extend to months or even years, when disease burden as well as carer burden is high and practical, emotional and spiritual coping strategies are no longer sufficient.

### Clinical Applications

In the late stages of Parkinson’s disease, people do report areas where they find meaning, such as family, partnership, spirituality and satisfaction. However, the fulfilment of their individual meaning in life areas is challenging, reflected by low satisfaction rates in these important categories.

Meaning in life and spiritual well-being negatively influence psychological distress in palliative care patients; this also plays a role in the development of a Wish to Hasten Death (WTHD). In a recent study on completed assisted suicides at a Swiss Right-to-Die organization, patients with Parkinsonian disorders made up for 7.2% and appeared to be overrepresented considering the prevalence of these diseases (Nuebling et al., 2021).

Only recently the importance of spiritual wellbeing in Parkinson’s disease has been shown to be related to general quality of life (Trang et al., 2020) and our study highlights spirituality as a core factor in meaning in life. Spiritual support should be a central part of multiprofessional care for people with neurodegenerative diseases and is not limited to religious questions. Appreciating spiritual needs of people with life-limiting diseases is an attitude more common in palliative care, than in general neurology. Since people with late-stage Parkinson’s disease often do not have access to long-term palliative care support, this indicates the importance of chaplains who can perform several roles in caring for people with Parkinson’s disease (Carey, 2012).

### Study Limitations

A drawback of our study is the limited availability of comparable data from healthy individuals from different European countries. We still decided to compare our data with the representative study (Fegg et al., 2007) with healthy German individuals, as this has been used as a reference group in studies with ALS, PSP and palliative care patients and allows some comparison with these patient groups. Even if we showed that age and age at disease onset do not have a significant effect on the Index of Weighted Satisfaction within our cohort of mostly elderly people with Parkinson’s disease, this could be of influence when comparing age groups of greater difference and also limits comparability to the cohort of healthy individuals of all age groups. Another limitation is that this study did not ask for people’s religious affiliation or how they identify them in terms of religious-spiritual belonging.

## Conclusions

Our study, which includes a very high number of people in late-stage of Parkinson’s disease from six European countries, contributes important information about the aspect of meaning in life. Given the lack of fulfilment in meaningful areas, a stronger focus on psychological and spiritual support for this group to strengthen their meaning in life is indispensable (Paal & Lorenzl, 2020).
